# The role of ^18^F-FCH PET/CT in patients with uremic hyperparathyroidism compared with ^99m^Tc-sestaMIBI SPECT/CT and ultrasonography

**DOI:** 10.1186/s13550-019-0583-9

**Published:** 2019-12-26

**Authors:** Yu Xue, Wenbo Li, Zhu Xia, Chengming Lei, Yiyi Cao, Zhengjie Wang, Hua Pang

**Affiliations:** Department of Nuclear Medicine, The First Affiliated Hospital of Chongqing Medical University, No. 1 Youyi Road, Chongqing, 400016 People’s Republic of China

**Keywords:** ^18^F-fluorocholine PET/CT, Uremic hyperparathyroidism, Hyper-functioning parathyroid gland, Parathyroid hormone

## Abstract

**Background:**

The aim of this study was to evaluate the diagnostic efficacy of ^18^F-fluorocholine (^18^F-FCH) PET/CT for uremic hyperparathyroidism (uHPT) compared to ^99m^Tc-sestaMIBI SPECT/CT and ultrasonography (US).

**Methods:**

A total of 17 uHPT patients with stage 5 chronic kidney disease (CKD) were prospectively enrolled. All patients underwent US, ^99m^Tc-sestaMIBI SPECT/CT, and ^18^F-FCH within 2 months and received surgical treatment. Visual and quantitative methods were used for image analyses. Intraoperative localization and postoperative histological results of the reference standard as well as the sensitivity, specificity, accuracy, positive predictive value (PPV), and negative predictive value (NPV) of the three modalities were analyzed using Pearson’s *χ*^2^ tests. In addition, the diagnostic efficacy of ^18^F-FCH PET/CT for uHPT was evaluated. The relationships between PET parameters and laboratory parameters were assessed using the Spearman correlation coefficient.

**Results:**

A total of 63 parathyroid hyperplasia lesions were resected in 17 uHPT patients, and 53 lesions were detected using ^18^F-FCH PET/CT with no false-positive results. The sensitivity, specificity, accuracy, PPV, and NPV were 84.13%, 100%, 86.49%, 100%, and 52.38%, respectively. In comparison, the corresponding values for ^99m^Tc-sestaMIBI SPECT/CT and US were 63.49%, 90.91%, 67.57%, 97.56%, and 30.30% and 61.90%, 81.82%, 64.86%, 95.12%, and 27.27%, respectively. The volume of hyper-functioning parathyroid glands was significantly different between lesions positive in ^18^F-FCH PET/CT and negative in ^18^F-FCH PET/CT (mean volume 1.36 ± 0.55 cm^3^ vs. 0.83 ± 0.26 cm^3^; *P* = 0.019). US misidentified intrathyroidal parathyroid hyperplasia as thyroid nodules in three patients, while ^18^F-FCH PET correctly identified the anatomy. No significant associations were observed between PET parameters and laboratory parameters in uHPT.

**Conclusion:**

^18^F-FCH PET/CT was more sensitive and accurate for uHPT than ^99m^Tc-sestaMIBI SPECT/CT and US, and had better preoperative diagnostic efficacy, particularly for lesions diagnosed as a thyroid nodule by US.

## Background

Uremic hyperparathyroidism (uHPT) is a common complication of end-stage chronic kidney disease (CKD), including secondary hyperparathyroidism (SHPT) and tertiary hyperparathyroidism (THPT) [1]. In CKD progression, both hyperphosphatemia and 1,25-dihydroxy vitamin D deficiency cause hypocalcemia and stimulate parathyroid hormone (PTH) secretion from the parathyroid gland, called SHPT [2]. SHPT patients remain in a stage of HPT 1 year after receiving a kidney transplantation, called THPT [3, 4]. Hypertrophic parathyroid glands of these patients develop into hyperplasia or adenoma that is not affected by serum levels of calcium and autonomously secrete excessive PTH [3, 4]. With the increasing prevalence of chronic diseases such as diabetes and hypertension, the overall incidence of CKD continues to rise. In addition, the incidence of uHPT is increasing, which seriously affects the prognosis of CKD patients [1].

For patients with uHPT, surgical management leads to superior survival and biochemical cure rates, as well as more favorable cost profiles compared to medical treatment [1, 5]. The indications for parathyroidectomy (PTX) in THPT are quite broad, including hypercalcemia, nephrocalcinosis, severe pruritus, low bone mineral density, and symptomatic hypercalcemia [6]. The 2017 Kidney Disease Improving Global Outcomes (KDIGO) guideline suggests that persistent parathyroid hormone (PTH) elevations over ninefold the upper limit of normal should be considered an indication for surgical management [7]. However, considering the poor physical condition of the uremic population, it is important for the surgeon to obtain information about parathyroid glands by imaging prior to PTX [1, 8]. Accurate preoperative gland localization can help surgeons rapidly identify the parathyroid gland during an operation and reduce the operative time. In addition, early detection of ectopic glands may help reduce the risk of recurrence by resection [1].

Routine preoperative gland localization imaging for PTX includes parathyroid scintigraphy with ^99m^Tc-sestaMIBI and ultrasonography (US) of the neck [9, 10]. However, no conventional imaging method shows great superiority at present. ^18^F-fluorocholine (FCH) PET, a common imaging modality used for prostate cancer, can be used to localize hyper-functioning parathyroid glands [11], and many studies have indicated that it has high diagnostic efficiency for primary hyperparathyroidism (PHPT) [9, 12–14]; nevertheless, no clinical study has only evaluated the diagnostic value of ^18^F -FCH PET for uHPT. In this study, the diagnostic performance of preoperative gland localization of ^18^F -FCH PET/CT for uHPT was investigated compared to ^99m^Tc-sestaMIBI SPECT/CT and US.

## Materials and methods

### Study subjects

This prospective study included 17 patients with uHPT who visited our hospital between December 2017 and January 2019. Patients were eligible for inclusion if they met the following criteria: clinically and biochemically diagnosed with SHPT or THPT, and candidacy for first PTX. We required patients to complete the scans within 2 months and receive surgical treatment within 2 months of ^18^F-FCH PET/CT imaging. Informed consent forms were signed by all patients. The study was approved by the medical ethics committee of our institution.

### Image acquisition

#### ^18^F-FCH PET/CT protocol

PET/CT images were acquired on a Philips Gemini TF 64 PET/CT scanner. Early images and late images of the neck and upper mediastinum region were acquired 10 min and 60 min after intravenous injection of 111–185 MBq (3–5 mCi) FCH (produced by Sumitomo accelerator of Japan with a radiochemical purity of > 95%), respectively. PET images were acquired for two bed positions (5 min/bed position). Low-dose CT images were obtained with a standardized protocol of 100 mA, 120 Kv, matrix size of 512 × 512, and a slice thickness of 2 mm. The MIP (maximum intensity projection) images and fusion images were obtained by computer iterative reconstruction and attenuation correction.

#### ^99m^Tc-sestaMIBI SPECT/CT protocol

Both ^99m^Tc-sestaMIBI dual-time planar images, MIP images, and ^99m^Tc-sestaMIBI SPECT/CT fusion images were obtained for all patients. Following an intravenous injection of 555–740 MBq (15–20 mCi) ^99m^Tc-sestaMIBI, early (20 min) and late (90 min) static images of 5 min of the neck and thorax were obtained using the GE Discovery 670 SPECT/CT with a low-energy high-resolution collimator (matrix 256 × 256, zoom level 1.45). SPECT/CT imaging was performed at the 90th minute immediately after obtaining static images for the neck and thorax. SPECT data were acquired over 360, yielding 60 projections at 15 s/projection. Low-dose CT images were obtained with a standardized protocol of 30 mA, 120 Kv, matrix size of 256 × 256, and a slice thickness of 5 mm.

#### US protocol

Each patient was examined in a supine position with the neck fully exposed. Experienced sonographers conducted transverse and longitudinal exploration of the anterior cervical region using a high-frequency linear transducer extending from the angle of the mandible to the sternal notch and inclined the probe to carefully observe the subclavian and sternal notch area. Doppler US images of suspected hypoechoic lesions were taken to evaluate blood flow signals.

### Image interpretation

The ^18^F-FCH PET/CT images were separately reviewed by two experienced nuclear specialists, as were the ^99m^Tc-sestaMIBI dual-phase and ^99m^Tc-sestaMIBI SPECT/CT images. Visual examination was performed to search for sites with higher focal tracer uptake in PET and SPECT images than the adjacent background activity and with a corresponding nodular lesion on CT. The size, maximum standard uptake value (SUVmax), and metabolic tumor volume (MTV; the volume of interest consisting of all spatially connected voxels within a fixed threshold of 40% of the SUVmax [15]) of the positive lesions were measured and used for semi-quantitative estimation. Lesions were localized anatomically to six regions: right upper, right lower, left upper, left lower thyroid, intrathyroidal, and ectopic (e.g., mediastinal).

With intraoperative localization and postoperative histological results as the reference standard, the imaging results were interpreted as follows: true-positive, location with regional tracer uptake as well as pathological confirmation of a hyper-functioning parathyroid gland; false-positive, location with regional tracer uptake and a histological result other than a hyper-functioning parathyroid gland; true-negative, location without regional tracer uptake and a histological result other than a hyper-functioning parathyroid gland; and false-negative, location without regional tracer uptake but with pathological confirmation of a hyper-functioning parathyroid gland.

### Surgery

All operations were performed by the same experienced surgeon. Patients underwent routine bilateral neck exploration and PTX, and some patients required parathyroid autotransplantation. Hematoxylin and eosin (HE) staining was carried out for histopathological analysis. PTH level was measured on the first morning after surgery; the procedure was considered successful if there was a decrease in the serum PTH value by 50% or more from the baseline value. Successful PTX in THPT patients was defined as maintaining normal serum levels of calcium for at least 6 months, regardless of whether PTH decreased [6].

### Statistical analyses

All statistical analyses were performed with software (IBM SPSS Statistics, version 25). Continuous variables are expressed as the mean ± standard deviation (SD). The sensitivity, specificity, accuracy, positive predictive value (PPV), and negative predictive value (NPV) of the three imaging modalities were analyzed statistically using Pearson’s *χ*^2^ tests with postoperative histology results as the gold standard. Student’s *t* test for parametric variables was applied to determine the significance of differences in lesion volumes between the two groups. The relationship between PET parameters and laboratory parameters was assessed using Spearman’s correlation coefficient. *P* value < 0.05 was considered significant.

## Results

### Surgery, histology results, and follow-up

The basic characteristics of the 17 enrolled patients (10 males, 7 females) are shown in Table 1, including 16 SHPT and 1 THPT. The reference normal range was 2.25–2.75 mmol/L for calcium and 12.0–88.0 pg/mL for PTH.

**Table 1:** Characteristics of the patients enrolled in the study

Parameter	Mean ± SD
uHPT	
Age (years)	50.82 ± 10.56
Serum calcium preoperative (mmol/L)	2.56 ± 0.25
ALP preoperative (U/L)	629.35 ± 611.23
BAP preoperative (μg/L)	103.38 ± 34.42
SUVmax	3.81 ± 1.46
SUVmean	1.63 ± 0.58
MTV (cm^3^)	1.39 ± 0.81
Total MTV (cm^3^)	4.25 ± 1.05
Total SUVmax	11.89 ± 2.66
SHPT	
PTH preoperative (pg/mL)	2191.93 ± 840.85
PTH decrease (%)	93.97 ± 9.86

A total of 68 lesions were resected, including 63 hyper-functioning parathyroid glands and 5 thyroid nodules in terms of the histological results. Interestingly, 1 patient had 3 hyperplastic parathyroid glands, 1 parathyroid adenoma, and a thyroid microscopic papillary carcinoma. In addition, 1 patient had 2, 3 patients had 3, and 12 patients had 4 hyperplastic glands. Four patients had thyroid nodules, including a thyroid cyst, a microscopic papillary thyroid carcinoma, and a nodular goiter, and two had nodular Hashimoto’s thyroiditis. On follow-up, all patients underwent successful surgery. Details of each patient are shown in Additional file 1: Table S1.

A total of 60 lesions were located behind or below the thyroid glands: 16 upper left, 17 lower left, 13 upper right, and 14 lower right. The other 3 hyperplastic lesions were located in the right lobe of the thyroid gland. The number of lesions localized by the 3 methods at different locations is shown in Additional file 1: Table S2.

### Diagnostic efficacy and superiority of ^18^F-FCH PET/CT

**Fig. 1:**
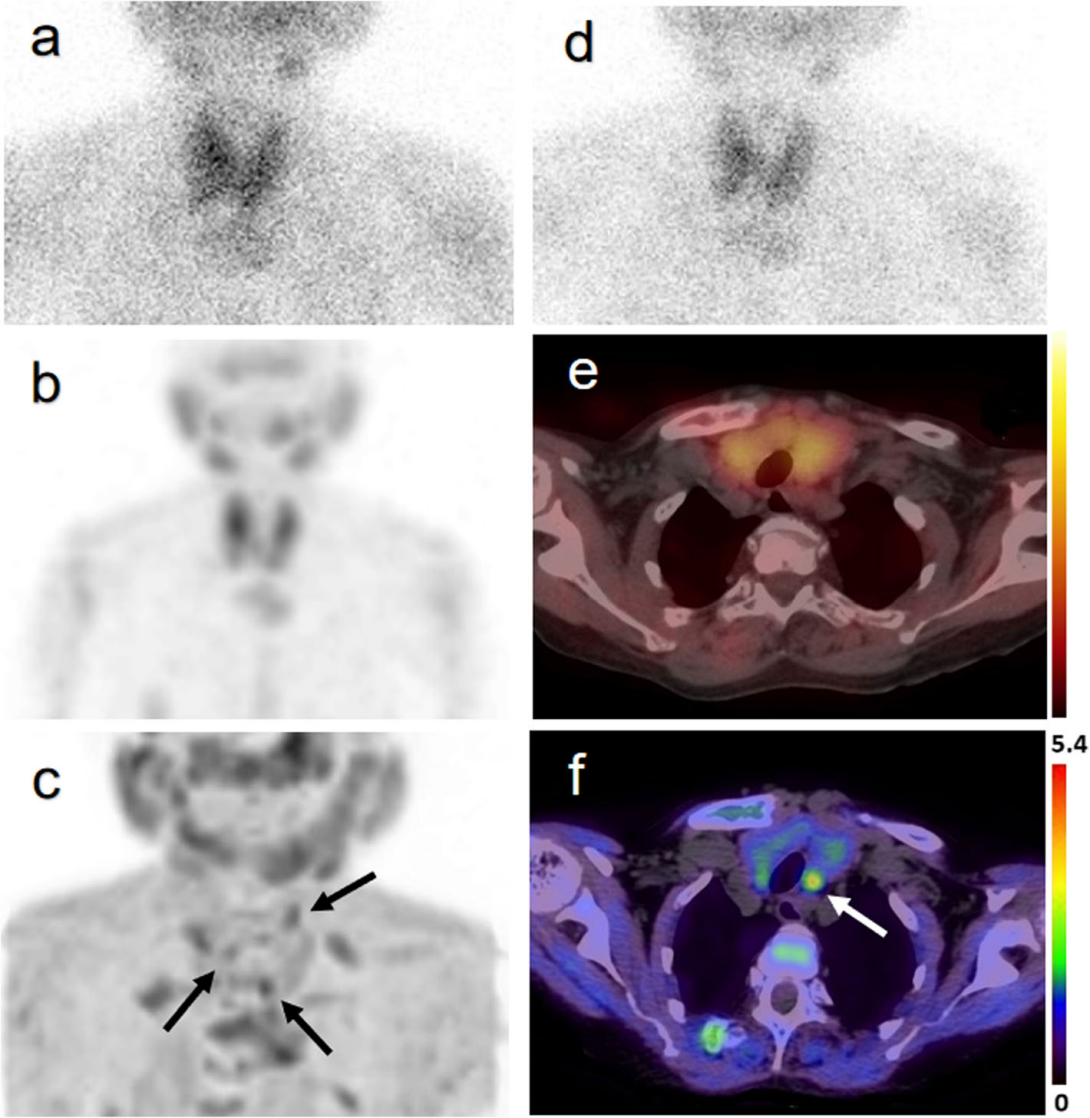
Images of a 62-year-old woman (case 11) with three parathyroid hyperplastic lesions. The early (**a**) and late (**d**) planar ^99m^Tc-sestaMIBI SPECT/CT images, MIP (maximum intensity projection) images of ^99m^Tc-sestaMIBI SPECT/CT (**b**), and fusion image (**e**) cannot present all three lesions clearly because of Hashimoto’s thyroiditis. Although the MIP image of ^18^F-FCH PET (**c**) is unclear due to renal osteopathy, the lesion is visible in the image (arrows). An ^18^F-FCH PET/CT fusion image (**f**) shows homogeneous tracer uptake behind the left lobe of the thyroid gland (arrow)

Of the 63 parathyroid lesions removed by surgery, ^18^F-FCH PET/CT correctly localized 53 lesions with no false-positive results. All lesions were visible in PET/CT images at both imaging times. The ^99m^Tc-sestaMIBI SPECT/CT allowed for correct localization of 40 lesions with 1 false-positive result. US allowed for correct localization of 39 lesions with 2 false-positive results. Additional file 1: Table S3 shows true-positive, true-negative, false-positive, and false-negative results of each imaging modality. The performance of ^18^F-FCH PET imaging was superior to that of the other two conventional imaging methods. Details are shown in Table 2. An example of a lower left hyperplastic gland localized on ^18^F-FCH PET/CT and with poor uptake on ^99m^Tc-sestaMIBI SPECT/CT is shown in Fig. 1.

**Table 2:** Diagnostic performance of ^18^F-fluorocholine PET/CT and conventional imaging modalities

	Sensitivity (%)	Specificity (%)	Accuracy (%)	PPV (%)	NPV (%)
^18^F-FCH PET/CT	84.13^*,**^	100	86.49^*,**^	100	52.38
^99m^Tc-sestaMIBI SPECT/CT	63.49	90.91	67.57	97.56	30.3
Ultrasonography	61.9	81.82	64.86	95.12	27.27

^*^The lesion-based sensitivity and accuracy of ^18^F-FCH PET/CT were significantly higher than those of ^99m^Tc-MIBI SPECT/CT (*P* = 0.008; *P* = 0.006)

^**^The lesion-based sensitivity and accuracy of ^18^F-FCH PET/CT were significantly higher than those of ultrasonography (*P* = 0.005; *P* = 0.006)

**Fig. 2:**
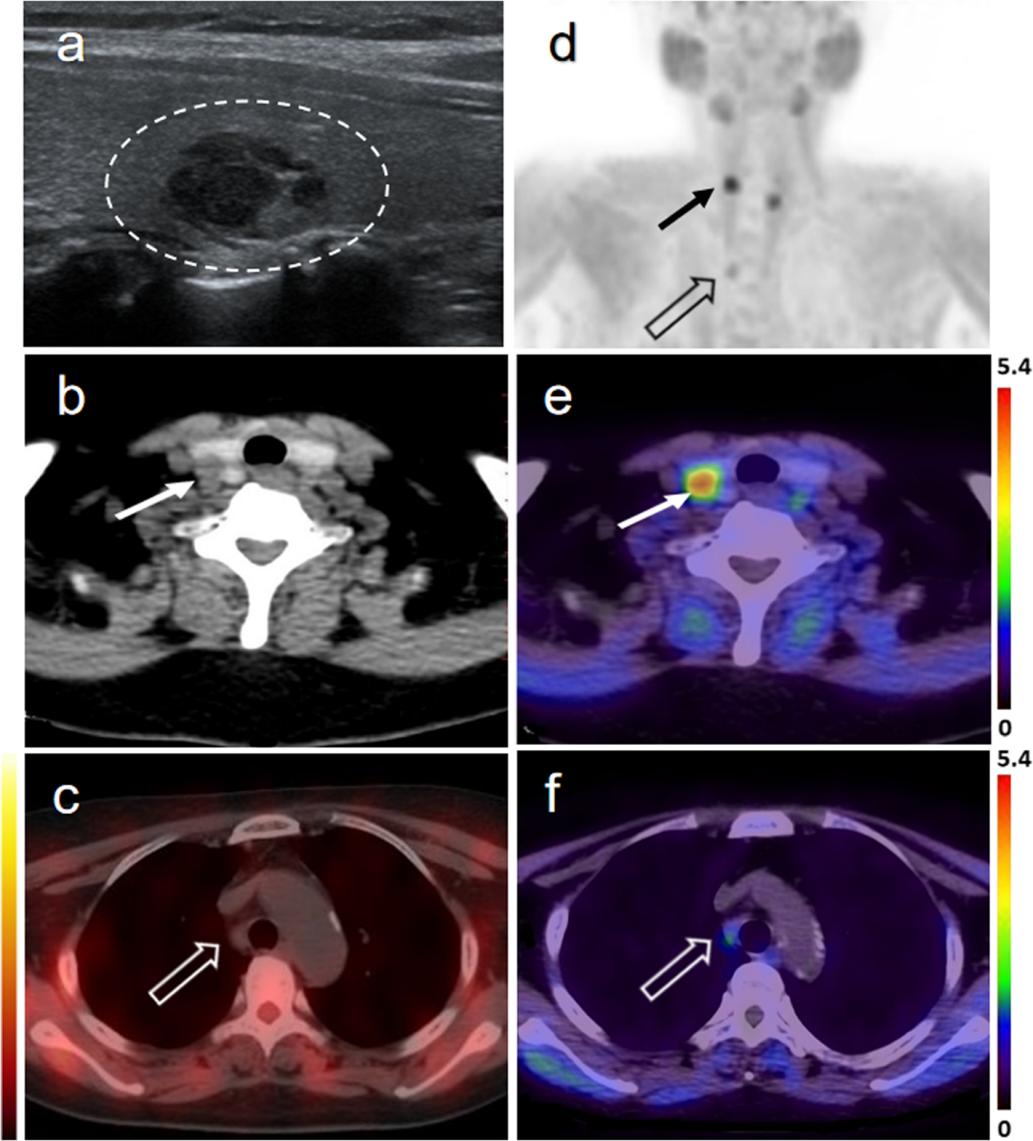
Images from a 61-year-old woman with multiple parathyroid hyperplasia, one with a slightly ectopic (in the middle portion of the right thyroid gland) localization. US misdiagnosed this lesion as a thyroid nodule (dotted circle) (**a**). The lesion is well delineated on the MIP image of ^18^F-FCH PET (**d**), axial CT (**b**), and PET/CT fusion images (**e**) (arrows). A heterogeneous low-density nodule in the upper mediastinum is well-delineated on the MIP image of ^18^F-FCH PET (**d**) and PET/CT fusion images (**f**) (hollow arrows), but no abnormal tracer uptake was observed on a ^99m^Tc-sestaMIBI SPECT/CT image (**c**) (hollow arrow)

As presented in Table 3, volumes of the hyper-functioning parathyroid glands were significantly different between lesions positive in ^18^F-FCH PET/CT and negative in ^18^F-FCH PET/CT. Lesions positive in ^18^F-FCH PET/CT and positive in ^99m^Tc-sestaMIBI SPECT/CT were larger than lesions positive in ^18^F-FCH PET/CT and negative in ^99m^Tc-sestaMIBI SPECT/CT, but the difference was not significant. In all, 3 of 63 lesions were located in the right lobe of the thyroid gland. US images of intrathyroidal parathyroid hyperplasia in all 3 patients were misdiagnosed as thyroid nodules; 1 of 3 lesions presented with true-positive findings in ^99m^Tc-MIBI SPECT/CT images and all 3 lesions presented with increased uptake in ^18^F-FCH PET images (mean SUVmax 5.87, mean MTV 2.2 cm^3^). An example of intrathyroidal parathyroid hyperplasia is shown in Fig. 2(a, b, d, e).

**Table 3:** Histopathological volume in hyper-functioning parathyroid glands from patients who tested positive/negative in PET/CT and SPECT/CT

	Size (mm^3^), mean ± SD	*P* value
PET/CT-positive	1.36 ± 0.55 (*n* = 53)	
PET/CT-negative	0.83 ± 0.26 (*n* = 10)	0.019^***^
PET/CT-positive and SPECT/CT-positive	1.52 ± 0.56 (*n* = 35)	
PET/CT-positive and SPECT/CT-negative	1.05 ± 0.36 (*n* = 18)	0.097

***Volumes of the hyper-functioning parathyroid glands positive in ^18^F-FCH PET/CT were significantly different from those negative in ^18^F -FCH PET/CT

### Relationship between PET parameters and preoperative laboratory parameters

Excessive PTH secretion in uHPT patients can enhance osteolysis, and the activities of alkaline phosphatase in serum (ALP) and bone (BAP) increase moderately or severely [16]. However, in this study, no significant association was observed among the sum of SUVmax, the sum of MTV, preoperative PTH, ALP, and BAP levels across the patients. Correlation coefficients between PET parameters and preoperative laboratory parameters are shown in Additional file 1: Table S4.

### Ectopic abnormal uptake of FCH

An inhomogeneous low-density nodule in the mediastinum showed moderate physiological tracer uptake on ^18^F-FCH PET/CT imaging, but no tracer uptake was observed on ^99m^Tc-sestaMIBI SPECT/CT imaging and the nodules were not operated on Fig. 2(c, d, f). According to the PTH level at the postoperative follow-up exam, the nodule was considered an enlarged lymph node. Therefore, ectopic abnormal uptake of FCH may be a false-positive finding. However, this study evaluated the true- or false-positive results based on pathological results. In the absence of surgical exploration of the lesion, we did not define the lesion as a false-positive location, but excluded it.

## Discussion

In this study, we prospectively evaluated the performance of ^18^F-FCH PET/CT imaging in uHPT patients. The crucial findings of the study are summarized as following. First, ^18^F-FCH PET/CT imaging has high sensitivity, specificity, accuracy, and PPV for hyper-functioning parathyroid gland localization. The lesion-based sensitivity and accuracy of ^18^F-FCH PET/CT were significantly higher than those of ^99m^Tc-sestaMIBI SPECT/CT and US. Second, ^18^F-FCH PET/CT is superior to US in identifying intrathyroidal parathyroid hyperplasia. Third, no significant associations were observed between PET parameters and laboratory parameters in SHPT.

After a clinical study [11] in 2014 showed that parathyroid adenoma in patients with prostate cancer were observed in ^18^F-FCH PET imaging, some studies [9, 17–19] were published suggesting that ^18^F-FCH PET could be used not only for hyper-functioning gland localization but also showed high sensitivity in patients with PHPT compared to conventional imaging methods, reaching as high as 92–96% in per-lesion analyses. PHPT is mostly caused by a solitary parathyroid adenoma [20], and SHPT and THPT patients often present with multiple parathyroid hyperplasia of varying sizes [1, 4, 20]. A previous study [21] reported a strong positive correlation between SUVmax and preoperative PTH levels in PHPT patients. However, the PTH levels of uHPT were generally much higher than those of PHPT. So, the excellent diagnostic efficacy of ^18^F-FCH PET cannot be extrapolated easily to uHPT patients. Our study is the first study which focuses only on uHPT. Because most lesions were visualized in both early and late images, we did not analyze the diagnostic performance of the two phases separately.

Our results indicate a sensitivity of 84.13% (per-lesion analyses) for ^18^F-FCH PET/CT for the detection of hyper-functioning parathyroid glands in patients with uHPT, slightly below that of PHPT as reported in previous studies [9, 17–19]. This may be because uHPT patients often present with multiple glandular disorders, and some smaller lesions had no obvious tracer uptake [22]. Although some studies have reported greater uptake of FCH in parathyroid adenomas than in hyperplastic lesions for PHPT patients [18, 23], it remains unclear whether the lower sensitivity of ^18^F-FCH PET/CT for uHPT than PHPT is attributable to pathological features. Overall, in our study, ^18^F-FCH PET had a higher sensitivity and accuracy compared to ^99m^Tc-sestaMIBI SPECT/CT and US in uHPT patients.

Alharbi et al. [21] reported a strong positive correlation between SUVmax and preoperative PTH levels and between adenoma background ratio (ABR) and preoperative PTH levels. Nevertheless, we did not observe a significant association between PET parameters and preoperative laboratory parameters in 16 SHPT patients. This difference between studies may be due to the fact that PTH levels of SHPT patients were generally higher than those of PHPT patients included in the previous study (mean PTH level 2191.93 ± 840.85 pg/mL vs. 122.4 ± 49.9 pg/mL).

In our study, ^18^F-FCH PET/CT showed superior diagnostic performance compared to ^99m^Tc-sestaMIBI SPECT/CT in detecting hyperparathyroidism. In ^18^F-FCH PET/CT true-positive findings, some lesions were not detected by MIBI, which may be associated with the small gland size. Previous studies have investigated lesions closely associated with thyroid location and/or patients with adenoma with abnormal washout patterns, who had undergone thyroid surgery, or with multiglandular disorder syndrome (MGDS), which may have resulted in the reduced sensitivity of ^99m^Tc-sestaMIBI [24]. Furthermore, compared to ^99m^Tc-sestaMIBI SPECT/CT, ^18^F-FCH PET/CT has significant technical advantages such as higher spatial resolution, shorter imaging time, and lower radiation exposure [12, 25, 26].

For PHPT patients, the sensitivity of US for detecting enlarged parathyroid glands ranges from 70 to 100% in experienced hands [27]. Nevertheless, in the presence of multinodular goiter and multiple parathyroid lesions, the sensitivity decreases from 47 to 84% in unskilled hands [27]. Patients with uHPT often have multiple lesions, which in turn decreases the sensitivity of US. Intrathyroidal parathyroid adenomas are easily missed by US. A previous study [28] showed that a hyperechoic line is produced by the strong reflection of US in the layer where the parathyroid gland is histologically separated from thyroid tissue, which represents the very thin capsules of both the parathyroid and thyroid glands. Although a hyperechoic line is the main sign of intrathyroidal parathyroid adenoma, it is difficult to identify and is often ignored by operators [28]. In this study, ^18^F-FCH PET correctly located three instances of intrathyroidal parathyroid hyperplasia, which were misdiagnosed as thyroid nodules by US, and the SUVmax of intrathyroidal hyperplasia was higher than that of other parathyroids in the same patient. ^18^F-FCH PET is a functional imaging modality that helps to distinguish parathyroid glands from thyroid nodules, although benign and malignant thyroid nodules may manifest regional ^18^F-FCH uptake [29–32]. ^18^F-FCH PET is still superior to US in this respect if we comprehensively analyze the PTH, SUVmax, and tracer uptake of other parathyroid glands. In addition, combined with the high spatial resolution of PET/CT, it is conducive for the visualization of parathyroid glands.

Although ^18^F-FCH PET is highly sensitive to hyper-functioning parathyroid glands, false-positive and false-negative results should be considered. One meta-analysis [33] showed that decreased volumes of individual glands and pathological characteristics, such as a low number of functional adenomatoid cells or oxyphilic cells, may result in false-negative results in ^18^F-FCH PET. Calabria et al. [34] showed that enlarged lymph nodes, thymoma, adrenal adenoma, meningioma, sarcoidosis, colon cancer, bladder cancer, and multiple myeloma may manifest with abnormal FCH uptake. Therefore, when identifying lesions with abnormal FCH uptake, we should explore the anatomical site to distinguish it from enlarged lymph nodes and thymoma to reduce false-positive results and identify small lesions. More importantly, we should pay attention to distinguishing thyroid nodules and intrathyroidal parathyroids and study the detailed morphological and metabolic differences in future work.

## Conclusion

^18^F-FCH PET/CT was more sensitive and accurate for uHPT than ^99m^Tc-sestaMIBI SPECT/CT and US and had better preoperative diagnostic efficacy, particularly for lesions diagnosed as thyroid nodules by US.

## Supplementary information

**Additional file 1: Table S1**. Detailed information of the patients enrolled in the study. **Table S2**. Locations of histologically verified hyper-functioning parathyroid glands on 18F-FCH PET/CT, 99mTc-sestamibi SPECT/CT and ultrasonography images. **Table S3**. True-positive, true-negative, false-positive and false-negative results of 18F-FCH PET/CT, 99mTc-sestamibi SPECT/CT and ultrasonography. **Table S4**. The correlation between PET parameters of 18F-FCH and preoperative laboratory level of patients with uremic hyperparathyroidism.

## Data Availability

Most data generated or analyzed during this study are included in this published article and its supplementary information files. The datasets generated or analyzed during the current study are available from the corresponding author on reasonable request, if you are confused about some of the data.

## Abbreviations

^18^F-FCH: Fluorine 18- fluorocholine
^99m^Tc-sestaMIBI: Technetium 99m-sestamibi
ABR: Adenoma background ratio
ALP: Alkaline phosphatase
BAP: Bone alkaline phosphatase
CKD: Chronic kidney disease
KDIGO: Kidney Disease Improving Global Outcomes
MGDS: Multiglandular disorder syndrome
MTV: Metabolic tumor volume
PET/CT: Positron emission tomography combined with computerized tomography
PHPT: Primary hyperparathyroidism
PTH: Parathyroid hormone
PTX: Parathyroidectomy
SD: Standard deviation
SHPT: Secondary hyperparathyroidism
SPECT/CT: Single-photon emission computed tomography combined with computerized tomography
SUVmax: Maximum standard uptake value
THPT: Tertiary hyperparathyroidism
uHPT: Uremic hyperparathyroidism
US: Ultrasonography
