# Supplementary material for: The role of 18F-FCH PET/CT in patients with uremic hyperparathyroidism compared with 99mTc-sestaMIBI SPECT/CT and ultrasonography
Source: EJNMMI Res. 2019 Dec 26;9:118. doi: 10.1186/s13550-019-0583-9 (PMC6933043; doi:10.1186/s13550-019-0583-9)
Supplement: Supplementary file 1 — Additional file 1: Table S1. Detailed information of the patients enrolled in the study. Table S2. Locations of histologically verified hyper-functioning parathyroid glands on 18F-FCH PET/CT, 99mTc-sestamibi SPECT/CT and ultrasonography images. Table S3. True-positive, true-negative, false-positive and false-negative results of 18F-FCH PET/CT, 99mTc-sestamibi SPECT/CT and ultrasonography. Table S4. The correlation between PET parameters of 18F-FCH and preoperative laboratory level of patients with uremic hyperparathyroidism. [file 13550_2019_583_MOESM1_ESM.docx]

**Additional file**

**Title**

The role of ^18^F-FCH PET/CT in patients with uremic hyperparathyroidism compared with ^99m^Tc-sestamibi SPECT/CT and ultrasonography

**Journal**

EJNMMI Research

**Authors**

Yu Xue^1^, Wenbo Li^1^, Zhu Xia^1^，Chengming Lei^1^, Yiyi Cao^1^，Zhengjie Wang^1^*, Hua Pang^1^*

**Author Affiliations**

1. Department of Nuclear Medicine, the First Affiliated Hospital of Chongqing Medical University, Chongqing, China

**Correspondence Information**

Dr. Zhengjie Wang, Department of Nuclear Medicine, the First Affiliated Hospital of Chongqing Medical University, No.1 Youyi Road, Chongqing, P.R. China, 400016

*E-mail address* [bkzg1234@163.com](mailto:bkzg1234@163.com)

*Tel* 89012386

*Fax* 00862389012386

Prof. Hua Pang, Department of Nuclear Medicine, the First Affiliated Hospital of Chongqing Medical University, No.1 Youyi Road, Chongqing, P.R. China, 400016

*E-mail address* [phua1973@163.com](mailto:phua1973@163.com)

*Tel* 89012386

*Fax* 00862389012386

**First author’s information**

Yu Xue, Department of Nuclear Medicine, the First Affiliated Hospital of Chongqing Medical University, No.1 Youyi Road, Chongqing, P.R. China, 400016

*E-mail address* [xyuu1994@163.com](mailto:xyuu1994@163.com)

**SUPPLEMENTAL TABLES**

**Table S1** Detailed information of the patients enrolled in the study

| Patient | | | Preoperative | | Location of Parathyroid | | | Postoperative | | |
| --- | --- | --- | --- | --- | --- | --- | --- | --- | --- | --- |
| NO. | Sex | Age(y) | Ca(mmol/L) | PTH（Pg/ml） | ^18^F-FCH | ^99m^Tc-sestamibi | Ultrasonography | Actual Location | Pathologycal Results | PTH（Pg/ml） |
| 1 | M | 38 | 2.47 | 2391.2 | LL,RU,RL | LL,RU,RL | LL,RU | LU,LL,RU,RL | 4 PH | 6.6 |
| 2 | M | 54 | 2.39 | 1322.3 | LU,RU,RL | LU,LL,RU,RL | LU,RU,RL | LU,LL,RU,RL | 4 PH | 343.9 |
| 3 | F | 61 | 2.53 | 1659.7 | LU,LL,**RT** | LL,RT | LU,LL | LU,LL,RT | 3 PH | 4.0 |
| 4 | M | 47 | 2.64 | 2782.5 | LU,LL,RU,RL | LL,RL | LL,RL | LU,LL,RU,RL | 4 PH | 6.0 |
| 5 | M | 64 | 2.25 | 1248 | LU,LL,RU | LL,RU | LL,RU | LU,LL,RU | 3 PH | 35.6 |
| 6 | M | 46 | 2.88 | 3347.1 | LU,RU | LU,LL,RU,RL | LU,RU | LU,LL,RU,RL | 4 PH | 986.2 |
| 7 | M | 47 | 2.81 | 224.2 | LL,RL | LL,RL | LL,RL | LL,RL | 2 PH | 155.7 |
| 8 | F | 40 | 2.63 | 3342.0 | LU,LL,RU,RL | LU,LL,RU,RL | LU,LL,RU,RL | LU,LL,RU,RL | 4 PH | 174.9 |
| 9 | F | 40 | 2.53 | 2339.3 | LU,LL,RU,RL | LL,RL | LU,LL,RU,RL | LU,LL,RU,RL | 4PH;RT-thyroid cyst | 38.0 |
| 10 | F | 50 | 2.69 | 1341.8 | LL,RL | LU,LL,RL | LU,LL,RU,RL | LU,LL,RU,RL | 4 PH | 4.9 |
| 11 | F | 62 | 2.83 | 2211 | LU,LL,RL | LL | RL | LU,LL,RL | 3 PH;RT-NHT | 438.7 |
| 12 | M | 47 | 2.01 | 3342.0 | LU,LL,RU,RL | LU,RL | LU,RL | LU,LL,RU,RL | LU,RU,RL-PH;LL-PA;RT-mPTC | 9.0 |
| 13 | M | 43 | 2.48 | 3230.0 | LL,RU | LL,RL | LU | LU,LL,RU,RL | 4 PH | 3.3 |
| 14 | M | 66 | 2.48 | 2030.4 | RL,**RT** | RL | RL | LU,LL,RT,RL | 4 PH | 26.2 |
| 15 | F | 63 | 2.83 | 2152.9 | LU,LL,RU,**RT** | LU,LL,RU | LL | LU,LL,RU,RT | 4 PH | 6.1 |
| 16 | F | 63 | 2.52 | 747.7 | LU,LL,RU,RL | RL | LU,LL,RU,RL | LU,LL,RU,RL | 4 PH;LT-nodular goiter | 4.3 |
| 17 | M | 33 | 2.32 | 1583.0 | LU,LL,RU,RL | LU,LL | LU,LL | LU,LL,RU,RL | 4 PH | 123.9 |

LU=Left upper thyroid, LL=Left lower thyroid, RU=Right upper thyroid, RL=Right lower thyroid, LT= Left lobe of thyroid, RT= Right lobe of thyroid.

PH=parathyroid hyperplasia, PA=parathyroid adenoma, NHT=Nodular Hashimoto’s thyroiditis, mPTC=microscopic papillary thyroid carcinoma.

Case 7 was patient with tertiary hyperparathyroidism (THPT) and the other patients were secondary hyperparathyroidism (SHPT). Case 12 had three hyperplastic parathyroid glands, one parathyroid adenoma (LL), and a microscopic papillary thyroid carcinoma. Four patients had thyroid nodules, including a thyroid cyst (case 9), two Nodular Hashimoto’s thyroiditis (case 11), a mPTC (case 12), and a nodular goiter (case 16).

**Table S2** Locations of histologically verified hyper-functioning parathyroid glands on ^18^F-FCH PET/CT, ^99m^Tc-sestamibi SPECT/CT and ultrasonography images

|  | ^18^F-FCH | ^99m^Tc-sestamibi | Ultrasonography | Surgical |
| --- | --- | --- | --- | --- |
| LU | 12 | 7 | 10 | 16 |
| LL | 14 | 14 | 11 | 17 |
| RU | 12 | 6 | 8 | 13 |
| RL | 12 | 12 | 10 | 14 |
| RT | 3 | 1 | 0 | 3 |
| Total | 53 | 40 | 39 | 63 |

LU=Left upper thyroid, LL=Left lower thyroid, RU=Right upper thyroid, RL=Right lower thyroid, RT= Right lobe of thyroid.

There was no significant difference in the number of lesions behind or below the bilateral thyroid glands detected by the three modalities. However, ^18^F-FCH PET/CT is superior to ultrasonography in localizing intrathyroidal parathyroid hyperplasia.

**Table S3** True-positive, true-negative, false-positive and false-negative results of ^18^F-FCH PET/CT, ^99m^Tc-sestamibi SPECT/CT and ultrasonography

|  | True-positive | False-positive | True-negative | False-negative |
| --- | --- | --- | --- | --- |
| ^18^F-FCH PET/CT | 53 | 0 | 11 | 10 |
| ^99m^Tc-sestaMIBI SPECT/CT | 40 | 1 | 10 | 23 |
| Ultrasonography | 39 | 2 | 9 | 24 |

In this study, it was assumed that each patient had four parathyroid glands: upper left, upper right, lower left, and lower right. If imaging localized two parathyroid glands to the same region, we assumed that the patient had five parathyroid glands.

**Table S4** The correlation between PET parameters of ^18^F-FCH and preoperative laboratory level of patients with uremic hyperparathyroidism

|  | Total SUVmax | Total MTV |
| --- | --- | --- |
| PTH preoperative | R=0.104,P=0.691 | R=0.295, P =0.251 |
| ALP preoperative | R=0.228, P =0.379 | R=0.222, P =0.392 |
| BAP preoperative | R=0.481, P =0.070 | R=0.206, P =0.461 |

No significant association was observed between the sum of maximum standardized uptake value (SUVmax) and preoperative parathyroid hormone (PTH), alkaline phosphatase in serum (ALP) and bone (BAP), between the sum of metabolic tumor volume (MTV) and preoperative PTH, ALP, BAP across the patients.
